# Perfectionistic concern profile as a risk factor for suicide-related behaviour in adolescents: Results from the EPISAM-School Study

**DOI:** 10.1192/j.eurpsy.2025.332

**Published:** 2025-08-26

**Authors:** A. De La Torre-Luque, P. Diaz-Carracedo, A. Garcia-Ramos, W. Ayad-Ahmed, A. Sanchez, S. Doval, C. Perez-Guerra, A. Pemau, L. J. Gonzalez-Agudelo

**Affiliations:** 1 Complutense University; 2 CIBERSAM ISCIII; 3 Hospital Clinico San Carlos, Madrid; 4 Barcelona University, Barcelona; 5 Autonomous University of Madrid, Madrid, Spain

## Abstract

**Introduction:**

Adolescents are at increased risk of developing suicide-related behaviour (SRB). Varying contributing factors may play an important role across the different forms of SRB. Perfectionistic concerns may become a cognitive moderator influencing volitional moderators (e.g., non-suicidal self-harm, NSSH) and suicidal ideation escalation.

**Objectives:**

To identify profiles of perfectionistic concerns in a community sample of adolescents. Also, to study the relationship between suicide-related outcomes and NSSH, according to perfectionism profile.

**Methods:**

A sample of 1,526 adolescents (54.3% female; *M*= 13.81 years, *SD*= 1.28) participated in our study. A wide range of SRB and motivational and volitional risk factors were evaluated in school settings. Six types of perfectionistic concerns, assessed by the Frost Multidimensional Perfectionism Scale (FMPS), were used to identify perfectionism profiles, through latent profile analysis.

**Results:**

Almost one in five adolescents (19.5%) showed SRB risk and more than one in three adolescents (35.1%) engaged in NSSH in the last year. Five profiles of perfectionism were identified (Figure 1). The profile featured by higher concerns across perfectionistic domains (7.2% of participants) showed significant relationship with SRB risk (OR = 2.84) and suicidal ideation (OR = 1.22), in comparison to the minimal concern profile. On the other hand, the profile featured by high parental concerns (18.2% of adolescents) was associated with increased risk of ideation (OR = 2.75) and NSSH (OR = 1.51).

**Image 1:**

**Figure:**
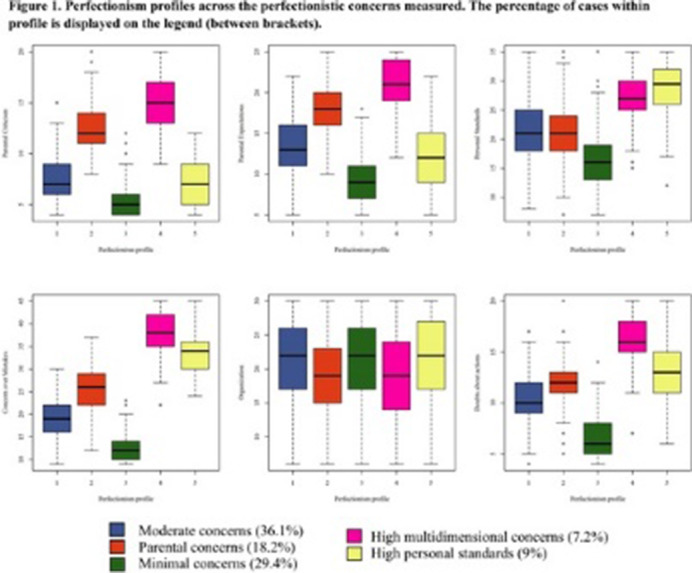

**Conclusions:**

Perfectionism may constitute a key risk factor for NSSH and SRB development. The promotion of prevention programmes to enhance cognitive regulation skills may help prevent suicide in adolescents.

**Disclosure of Interest:**

None Declared

